# 

**Published:** 1843-10-01

**Authors:** 


### BIBLIOGRAPHICAL RECORD.

1. The Student's Friend; or the Plea of Humanity and Common Sense against Surgical Operations for the Cure of Impediments of Speech. By JAMES WRIGHT, Esq. Second Edition, price 2s. 6d. Sherwood and Co. 56 pages.

2. Pharmaceutical Journal and Transactions. Edited by JACOB BELL. Vol. 3, No. 1. July, 1843. Price 1s.

3. Extracts from an unpublished Work on the Importation and Propagation of Plague and other Contagious Diseases. By Dr. FERGUSON, Inspector of Hospitals.

4. Advice to Wives on the Management of themselves, during the Period of Pregnancy, Labor, and Suckling. By P. HENRY CHAVASSE, M.R.C.S. London. Octavo, pp. 213. Second Edition. Longman and Co. 1843.

☞ *This Edition is a good deal improved. It is one of the most useful and honest little works which can be put into a female's hands.*

5. The Phrenological Journal, and Magazine of Moral Science. No. 76. July, 1843.

6. Report of the Managing Committee of the House of Recovery and Fever Hospital, Cork Street, Dublin, from the 1st of April, 1842, to the 1st of April, 1843—with the Medical Report, &c. By PATRICK HARKAN, M.D. Dublin, 1843.

7. Facts and Observations relative to the Influence of Manufactures upon Health and Life. By DANIEL NOBLE, M.R.C.S., &c. Octavo, pp. 81. Churchill, London, 1843.

8. Neurixpnology; or the Rationale of Nervous Sleep, considered in Relation with Animal Magnetism, &c. By JAMES BRAID, M.R.C.S. Octavo, pp. 260. Churchill, 1843.

9. Scarlatina and its Treatment on Homœopathic Principles. By JOSEPH BELLUOMINA. Bailliere, 1843.

10. A Medical Visit to Granberg, in April and May, 1843, for the purpose of investigating the Merits of the Water-cure Treatment. By Sir CHARLES SCUDAMORE, M.D. F.R.S. Octavo, pp. 105. Churchill, London, 1843.

---

11. Memoires de l'Academie Royale de Medecine. Tome Dixieme, quarto. Paris, Bailliere—Londres, Bailliere. 1843.

---

12. Statistical Report of One Hundred and Ninety Cases of Insanity, admitted into the Retreat, near Leeds, during Ten Years, from 1830 to 1840. By SAMUEL HARE, late Superintendant of the Institution.—(From the Provincial Medical Journal.)

☞ *A very able and highly important report.*

---

13. Letter to the Right Honorable Sir Robert Peel, Bart., on the Responsibility of Monomaniacs for the Crime of Murder. By JAMES STARK, M.D., F.R.S.E. Octavo, pp. 42, sewed. Stark and Co. Edinb. 1843.

---

14. The Iodated Waters of Heilbrunn in Bavaria, considered with respect to their Efficacy in the Treatment of Scrofulous, Cutaneous, and other Diseases. By Sir ALEX. DOWNIE, M.D., Physician to Her Majesty's Legation at Frankfort. Frankfort, 1843.

---

15. Cataract, and its Treatment, comprising an easy Mode of dividing the Cornea for its Extraction, &c. By JOHN SCOTT, Senior Surgeon to the Royal London Ophthalmic Hospital, Surgeon to the London Hospital. Churchill, London. Price 2s. 6d. 1843.

---

16. Forty-seventh Report of the Friendly Retreat, near York; or State of an Institution, near York, called the RETREAT, for Persons afflicted with Disorders of the Mind. 1843.

☞ *Mr. Thurnam's Report is, as might be expected, sensible and modest.*

---

17. Atlas illustrative of the Anatomy of the Human Body. By J. CRUVEILHIER, M.D. &c. Part 20, with Explanations. 2s. 6d. Bailliere, Paris and London.

---

18. Anatomico-Chirurgical Observations on Dislocations of the Astragalus. By THOMAS TURNER, Esq. M.R.C.S. Surgeon to the Royal Manchester Infirmary, &c. Octavo, pp. 138. 1843.

---

19. Thirteenth Report of the Belfast Asylum for the Insane. By ROBERT STEWART, M.D. Resident. 1843.

---

20. Some Account of the African Remittent Fever, which occurred in Her Majesty's Steam-ship WILBERFORCE on the River Niger, &c. By MORRIS PRITCHETT, M.D. Octavo, pp. 184. Churchill, London. 1843.

☞ *Forty-three cases of fever are minutely detailed, and the remainder of the volume is dedicated to remarks on the African fever and its causes. This will prove a useful companion to Dr. M'William's work for the African surgeon.*

---

21. Observations on Idiopathic Dysentery, as it occurs in Europeans in Bengal, particularly in reference to the Anatomy of that Disease. By WALTER RALEIGH, Surgeon of the Native Hospital of Calcutta. Pp. 150. Calcutta, 1843.

---

22. Pulmonary Consumption successfully treated with Naphtha. By JOHN HASTINGS, M.D. Senior Physician to the Blenheim Street Free Dispensary. Octavo, pp. 120.

---

23. Lectures on the Comparative Anatomy and Physiology of the Invertebrate Animals, delivered at the Royal College of Surgeons, in 1843. By RICHARD OWEN, F.R.S. Hunterian Professor to the College. From Notes taken by WM. WHITE COOPER, M.R.C.S., and revised by Professor OWEN. Illustrated by numerous Woodcuts. Octavo, pp. 392.

---

24. The Mosaic Account of the Unity of the Human Race, confirmed by the Natural History of the American Aborigines. By SAMUEL FORRY, M.D., Author of "The Climate of the United States, and its Endemic Influence."

☞ *A very ingenious, interesting, and learned performance.*

---

25. On Ankylosis, or Stiff-Joint. A Practical Treatise on the Contractions and Deformities resulting from Diseases of Joints. By W. J. LITTLE, M.D. &c. &c. Octavo, pp. 145. London, 1843.

---

26. Contributions on the Influence of Employments upon Health. By WILLIAM AUGUSTUS GUY, M.B. &c. &c.

27. On the Arrangement and Nomenclature of Mental Disorders. A Prize Essay, to which the Society for the Improvement of the Condition of the Insane awarded the Premium of Twenty Guineas, March 1843. By Henry Johnson, M.D. Edinburgh. Octavo, stitched, pp. 33.

---

28. Principles of Forensic Medicine. By William A. Guy, M.B. Professor of Forensic Medicine, King's College, London, Physician to King's College Hospital, &c. Part I. 12mo. pp. 184. London, 1843.

---

29. The Vital Statistics of Sheffield. By E. Calvert Holland, Esq. M.D., &c. &c. Pp. 260. London, 1843.

---

30. The Retrospect of Practical Medicine and Surgery, being a half-yearly Journal, containing a Retrospective View of every Discovery and Practical Improvement in the Medical Sciences. Edited by W. Braithwaite, Surgeon to the Leeds General Eye and Ear Infirmary, &c. 1843.

☞ *A capital little book.*

---

31. Report of the Directors of the Montrose Lunatic Asylum, Infirmary, and Dispensary, for the Year ending June 1, 1843.

☞ *Noticed elsewhere.*

---

32. On the Safety Lamps of M. Mnesler and Dr. Reid Clanny. From the Gates-head Observer.

---

33. Some Account of the Epidemic of Scarlatina, which prevailed in Dublin, from 1834 to 1842, inclusive. With Observations. By Henry Kennedy, A.B., M.B. &c. Dublin, 1843.

---

34. The Naturalist's Library, conducted by Sir William Jardine, Bart. Icthyology, Vol. VI. British Fishes, Vol. II. By Robert Hamilton, M.D. F.R.S.E. 1843.

---

35. The Natural History of the Birds of Great Britain and Ireland. Part IV. Natatories. Illustrated by Thirty-three coloured Plates, with Portrait and Memoir of Wilson. By Sir William Jardine, Bart. 1843.

---

36. A Pathological and Philosophical Essay on Hereditary Diseases, with an Appendix, &c. By Julius Henry Steinam, of the Royal Medical College, Berlin. 8vo, pp. 52. Simpkin and Co. 1843.

---

37. The New General Biographical Dictionary. Projected and partly arranged by the late Rev. Hugh James Rose, B.D., Principal of King's College, London, Part XX.

---

38. On the Arrangement and Nomenclature of Mental Disorders. A Prize Essay. By Henry Johnson, M.D. *Ed.*, Senior Physican to the Salop Infirmary, &c. Longman & Co. 1843. Pp. 33. Price 1s. 6d.

---

39. Lectures on the Practice of Physic, delivered at King's College, London. By Thos. Watson, M.D. Fellow of the Royal College of Physicians, Physician to the Middlesex Hospital, &c. Two Vols. 8vo. pp. 830—812. Parker, 1843.

